# Combining biomarkers with clinical risk factors to predict acute kidney injury after cardiopulmonary bypass surgery: An observational cohort study

**DOI:** 10.1097/MD.0000000000049765

**Published:** 2026-07-10

**Authors:** Xuanxuan Xu, Yuhao Liang, Shuaibo Shi, Huiling Si, Axue Cheng, Junlong Wang, Tao Liu, Hui Wang, Jie Hu

**Affiliations:** a Department of Anesthesiology (Henan Key Discipline in Anesthesiology, Luoyang Key Medical Cultivation Discipline), Luoyang Central Hospital, Luoyang, Henan, China; b Department of Anesthesiology, Luoyang Central Hospital Affiliated to Henan Medical University, Luoyang, Henan, China.

**Keywords:** cardiac surgery-associated acute kidney injury, cardiopulmonary bypass, neutrophil gelatinase-associated lipocalin, soluble urokinase-type plasminogen activator receptor, urinary matrix metalloproteinase-7

## Abstract

Cardiac surgery-associated acute kidney injury (CSA-AKI) is a prevalent complication subsequent to cardiopulmonary bypass (CPB) surgery. Timely diagnosis is essential for enhancing patient outcomes. Traditional diagnostic approaches that depend on serum creatinine and urine output frequently result in delays; therefore, there is a pressing necessity to discover sensitive and specific biomarkers. This study is the 1st investigation to concurrently assess the collective predictive capacity of soluble urokinase-type plasminogen activator receptor (suPAR [a systemic inflammation biomarker]), neutrophil gelatinase-associated lipocalin (NGAL [an early indicator of renal tubular damage]), and matrix metalloproteinase-7 (MMP-7 [a marker of Wnt pathway activation]) for CSA-AKI. In this single-center observational study, 116 patients undergoing CPB cardiac surgery were enrolled and categorized into AKI and non-AKI groups according to kidney disease improving global outcomes criteria. Levels of plasma suPAR, urinary NGAL, and urinary MMP-7 were dynamically monitored preoperatively and at 2 hours, 12 hours, 2 days, and 4 days after cardiac surgery. Preoperative plasma suPAR demonstrated significant predictive value for AKI risk prior to surgery, with an area under the curve (AUC) of 0.727. At 2 hours postoperatively, urinary NGAL (AUC = 0.807) and urinary MMP-7 (AUC = 0.805) were identified as the earliest postoperative predictive biomarkers. The combination of suPAR, NGAL, and MMP-7 significantly enhanced predictive performance (AUC = 0.902, sensitivity 90%, specificity 81.8%). Furthermore, a logistic regression model incorporating these biomarkers and CPB time achieved the highest diagnostic accuracy (AUC = 0.931, sensitivity 90%, specificity 84.8%). The simultaneous assessment of preoperative plasma suPAR, postoperative 2-hour urinary NGAL, and urinary MMP-7 markedly improves the diagnostic accuracy for CSA-AKI. Additionally, incorporating CPB time into a nomogram-based predictive model enhances early warning capabilities, which may support early risk stratification. However, further external validation is required before routine clinical implementation.

## 1. Introduction

Cardiac surgery-associated acute kidney injury (CSA-AKI) remains a common and serious complication following cardiopulmonary bypass (CPB), with a reported incidence as high as 30% to 40%.^[1]^ CSA-AKI is associated with significantly increased patient mortality, prolonged intensive care unit (ICU) stays, and higher healthcare costs. Approximately 2% to 3% of affected patients require renal replacement therapy (RRT), and those receiving RRT face a 28-fold increased risk of progressing to chronic kidney disease (CKD).^[2,3]^ The pathogenesis of CPB-associated AKI involves multiple factors, including ischemia-reperfusion injury, systemic inflammatory response, and hemodynamic instability.^[4]^ Current diagnosis of CSA-AKI relies on the kidney disease improving global outcomes (KDIGO) criteria,^[5]^ which are based on changes in serum creatinine (Scr) and urine output. However, Scr levels are influenced by factors such as age and muscle mass and typically rise only 48 to 72 hours after renal injury, leading to delayed diagnosis. Urine output can be affected by volume status and diuretic use. This diagnostic time lag often delays early intervention, thereby adversely affecting patient outcomes.^[6]^

Recent studies have identified several tubular injury-associated proteins as promising early biomarkers. Neutrophil gelatinase-associated lipocalin (NGAL) is a 25 kDa single-polypeptide protein that modulates iron metabolism by binding bacterial siderophores.^[7]^ NGAL levels in urine and blood increase significantly within 2 hours after tubular injury. It exerts a renoprotective role by regulating iron homeostasis and suppressing inflammation and demonstrates superior sensitivity compared to Scr.^[8]^

The soluble urokinase-type plasminogen activator receptor (suPAR) is an emerging biomarker with distinct advantages, including high stability in various body fluids such as blood and urine, and resistance to confounding by age, muscle mass, or medications. Elevated suPAR levels have been documented in cancer,^[9]^ autoimmune diseases,^[10,11]^ and infectious diseases.^[12]^ suPAR has been established as a strong predictor of AKI.^[13,14]^ International studies indicate a significant correlation between suPAR levels and the incidence of AKI after cardiac surgery,^[15,16]^ which is further supported by a meta-analysis of 9 studies confirming a positive correlation with AKI severity.^[17]^ Gungor et al systematic review of 6410 surgical patients^[18]^ confirmed a robust association between perioperative plasma suPAR and postoperative complications, with the link to AKI supported by moderate-certainty evidence, establishing AKI as the most clinically predictive perioperative complication for suPAR. The study also postulated that suPAR directly contributes to AKI pathophysiology by mediating oxidative stress and proinflammatory cytokine release. However, the diagnostic value of suPAR specifically in adult patients following CPB cardiac surgery within the Chinese population requires further validation.

Matrix metalloproteinase-7 (MMP-7) is a 30 kDa zinc/calcium-dependent endopeptidase involved in pathological processes via degradation of the extracellular matrix and activation of cytokines.^[19,20]^ Its expression is transcriptionally regulated by AP-1 or the Wnt/β-catenin pathway and posttranslationally modulated through proteolytic activation.^[21]^ Previous studies have shown significant upregulation of MMP-7 in both human and animal models of CKD.^[22]^ While expressed at low or undetectable levels in healthy kidneys, MMP-7 is markedly induced following kidney injury, including in AKI and CKD.^[23]^ Although MMP-7 has demonstrated biomarker potential in various renal pathologies, its predictive efficacy for AKI after adult CPB cardiac surgery needs prospective validation.

This study is the 1st to simultaneously evaluate the diagnostic value of plasma suPAR, plasma/urinary NGAL, and urinary MMP-7 measured at multiple time points, for predicting AKI after cardiac surgery. Furthermore, by integrating these biomarkers with clinical risk factors to construct a predictive model, we comprehensively assess their combined efficacy for the early prediction of AKI following CPB.

## 2. Methods

### 2.1. Study population

This observational cohort study consecutively enrolled 116 patients who underwent cardiac surgery with CPB at Luoyang Central Hospital between February 2023 and February 2024. The relevant data for research purposes were systematically retrieved and collated from February 9, 2023 to April 30, 2024. The study protocol was approved by the Institutional Ethics Committee (Approval No. LWLL-2023-02-09-01) and was registered with the Chinese Clinical Trial Registry (ChiCTR2500099878). Written informed consent was obtained from all participants. This study was conducted in accordance with the Declaration of Helsinki.

The inclusion criteria were as follows: age >18 years; scheduled for elective or urgent/emergent CPB surgery (urgent/emergent surgery was defined as cardiac surgery performed within 24 hours of diagnosis of life-threatening cardiac conditions); left ventricular ejection fraction ≥35%, cardiac function classification II to IV, and no myocardial infarction within the past month; no history of prior cardiac surgery; normal electrolyte levels and acid-base balance; no active infection or allergic diseases; and provision of written informed consent by the patient or their legal guardian. Patients meeting any of the following criteria were excluded: preexisting CKD or chronic dialysis; AKI present before surgery; comorbid renal tumors, nephrolithiasis, urinary tract obstruction, or chronic liver disease; history of septic shock, active infection, or autoimmune diseases; death or cardiac arrest occurring within 48 hours postoperatively; requirement for intra-aortic balloon pump support after surgery; need for concurrent noncardiac surgery; or incomplete clinical data.

According to the KDIGO criteria,^[5]^ enrolled patients were categorized into an AKI group and a non-AKI group based on the occurrence of CSA-AKI within 7 days after surgery. The diagnosis of CSA-AKI was based on both Scr and urine output criteria (meeting either of the 2): an increase in Scr by ≥0.3 mg/dL (≥26.5 μmol/L) within 48 hours; or an increase in Scr to ≥1.5 times the baseline value (known or presumed to occur within 7 days); or a urine output of <0.5 mL/kg/h for 6 consecutive hours. The baseline Scr was defined as the latest measured Scr value within 1 week before surgery for elective cases, and the 1st measured Scr value after admission (before surgery) for urgent/emergent cases.

### 2.2. Demographic and clinical characteristics

Data were extracted from the hospital’s electronic medical record system. Preoperative parameters included gender, age, body mass index, medical history, use of contrast agents, emergency surgery status, estimated glomerular filtration rate, blood urea nitrogen, Scr, white blood cell count (WBC), neutrophil percentage, hemoglobin, albumin, left ventricular ejection fraction, and other baseline values. Intraoperative records comprised operation time, CPB time, aortic cross-clamp time, intraoperative urine output, blood loss, and volume of red blood cell (RBC) transfusion. Postoperative outcomes included Scr levels, re-sternotomy, RRT, mortality, ICU stay, and total hospital stay.

Blood and urine samples were collected preoperatively and at 2 hours, 12 hours, 2 days, and 4 days postoperatively. Blood samples were collected into EDTA anticoagulant tubes, allowed to stand vertically at room temperature for 2 hours or at 4°C for up to 4 hours, and centrifuged at 2000 rpm for 20 minutes to separate plasma. Urine samples (5–10 mL of fresh urine) were collected and processed under identical standing and centrifugation conditions to obtain the supernatant. All samples were aliquoted, sealed, and stored at −80°C until analysis, avoiding repeated freeze-thaw cycles. Levels of plasma NGAL, plasma suPAR, urinary NGAL, and urinary MMP-7 were quantified using commercially available enzyme-linked immunosorbent assay kits (Manufacturer: Xiamen LunChangShuo Biotechnology Co., Ltd.; Catalog Numbers: ED-11771 for NGAL, ED-14806 for suPAR, and ED-10754 for MMP-7). The detection ranges were 2.5 to 80 ng/mL for suPAR, 3.75 to 120 ng/mL for NGAL, and 0.75 to 24 ng/mL for MMP-7. All measurements were performed in duplicate according to the manufacturer’s instructions. The intra-assay and inter-assay coefficients of variation were both <15%. Furthermore, all laboratory personnel conducting the assays were strictly blinded to the patients’ clinical data and AKI status. The Scr values were obtained from routine clinical testing.

### 2.3. Statistical analysis

Based on a prior study showing plasma suPAR had an area under the curve (AUC) of 0.69,^[17]^ sample size calculation (α = 0.05, δ = 0.1) determined 106 subjects were needed. We enrolled 128 patients, with 116 included in the final analysis. Statistical analyses used SPSS 25.0 (IBM Corporation) and R software version 4.2.3 (R Core Team). Data normality was assessed by the Kolmogorov–Smirnov test. Continuous variables are expressed as mean ± standard deviation for normally distributed data or median with interquartile range for non-normally distributed data. Comparisons between groups were performed using the Student *t* test or the Mann–Whitney *U* test, as appropriate. Categorical variables are shown as n (%) and compared by chi-square/Fisher exact test. For repeated measures biomarker data across different time points, nonparametric tests were employed for analysis: the Friedman test was used for within-group comparisons, while the Mann–Whitney *U* test was applied for between-group comparisons at each time point. The Bonferroni correction was utilized to adjust for multiple comparisons across different time points and biomarkers.

Biomarker prediction was evaluated by receiver operating characteristic curve analysis. Multivariable logistic regression identified risk factors and built a nomogram, validated internally via 1000 bootstrap samples. Discrimination was assessed by AUC, calibration by calibration curves, and clinical utility by decision curve analysis. *P* < .05 was considered significant.

## 3. Results

### 3.1. General information comparison

**Figure 1.**
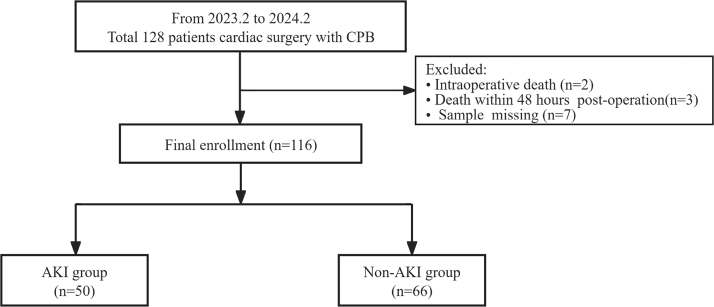
Flowchart of the study cohort. AKI = acute kidney injury, CPB = cardiopulmonary bypass.

A total of 128 patients were initially enrolled, of whom 12 were excluded for the following specific reasons: unavailable postoperative biomarker samples in 7 cases, intraoperative death in 2 cases, and death within 48 hours postoperatively in 3 cases. Thus, 116 patients were ultimately included in the study (Fig. 1). According to KDIGO criteria, 50 patients (43.1%, 50/116) developed CSA-AKI within 7 days after surgery. Intergroup comparisons revealed that the AKI group had significantly higher proportions of emergency surgery (*P* = .007), preoperative WBC (*P *= .007), operation time (*P* < .001), CPB time (*P* < .001), RBC transfusion volume (*P* = .028), mortality after 48 hours postoperatively (*P* = .008), and ICU stay (*P* < .001) compared to the non-AKI group. No statistically significant differences were observed in the remaining indicators (Table 1).

**Table 1:** Comparison of demographic and clinical data between AKI and non-AKI groups.

Variables	AKI (N = 50)	Non-AKI (N = 66)	*P* value
Preoperative parameters			
Age (yr)	56.7 ± 11.48	57.8 ± 10.4	.514
Sex (male)	36 (72)	49 (74)	.787
BMI (kg/m^2^)	24.27 ± 6.23	24.96 ± 4.29	.538
Hypertension (n)	28 (56)	41 (62)	.506
Diabetes mellitus (n)	4 (8)	7 (11)	.754
Emergency surgery (n)	30 (60)	23 (35)	.007
Contrast use (n)	17 (34)	15 (23)	.211
Albumin (g/L)	38.0 (35.1, 41.0)	39.9 (35.5, 41.5)	.427
Hb (g/L)	128 (115, 137)	128 (115, 140)	.675
WBC (×10^9^/L)	9.07 (6.518, 13.645)	6.390 (5.345, 9.240)	.007
BUN (mmol/L)	6.76 (5.69, 8.07)	6.0 (5.0, 7.9)	.192
Scr (μmol/L)	69.2 (54.1, 89.8)	68.0 (59.3, 91.0)	.800
eGFR (mL/min)	93.6 (73.3, 126.2)	87.5 (71.4, 124.0)	.674
NEUT (%)	73.5 (56.6, 87.3)	67.6 (59.2, 78.4)	.301
LVEF (%)	60 (58, 65)	60 (54, 64)	.186
Intraoperative parameters			
Operation time (min)	385 (311, 470)	285 (250, 375)	<.001
CPB time (min)	197 (168, 224)	143 (108, 179)	<.001
Cross-clamp time (min)	101 (87, 128)	93 (78, 124)	.143
RBC transfusion (U)	4 (0, 6)	0 (0, 4)	.028
Blood loss (mL)	700 (500, 1000)	500 (400, 800)	.050
Urine output (mL)	1100 (900, 1950)	1550 (1000, 2175)	.130
Postoperative parameters			
Re-sternotomy (n)	5 (10)	1 (1.52)	.083
RRT (n)	2 (4)	0 (0)	.184
Mortality (>48 h)	11 (22)	3 (5)	.008
ICU stay (d)	7.0 (6.0, 14.0)	4.5 (3.0, 8.0)	<.001
Hospital stay (d)	26.5 (19.3, 41.0)	29.0 (22.0, 41.0)	.371

Values are presented as mean ± standard deviation, median (IQR) or number (percentage).

ALB = albumin, AKI = acute kidney injury, BMI = body mass index, BUN = blood urea nitrogen, CPB = cardiopulmonary bypass, eGFR = estimated glomerular filtration rate, Hb = hemoglobin, ICU = intensive care unit, LVEF = left ventricular ejection fraction, NEUT% = neutrophil granulocyte%, RBC = Red blood cell, RRT = renal replacement therapy, Scr = serum creatinine, WBC = white blood cell.

### 3.2. Temporal changes of biomarkers in the 2 patient groups

**Figure 2.**
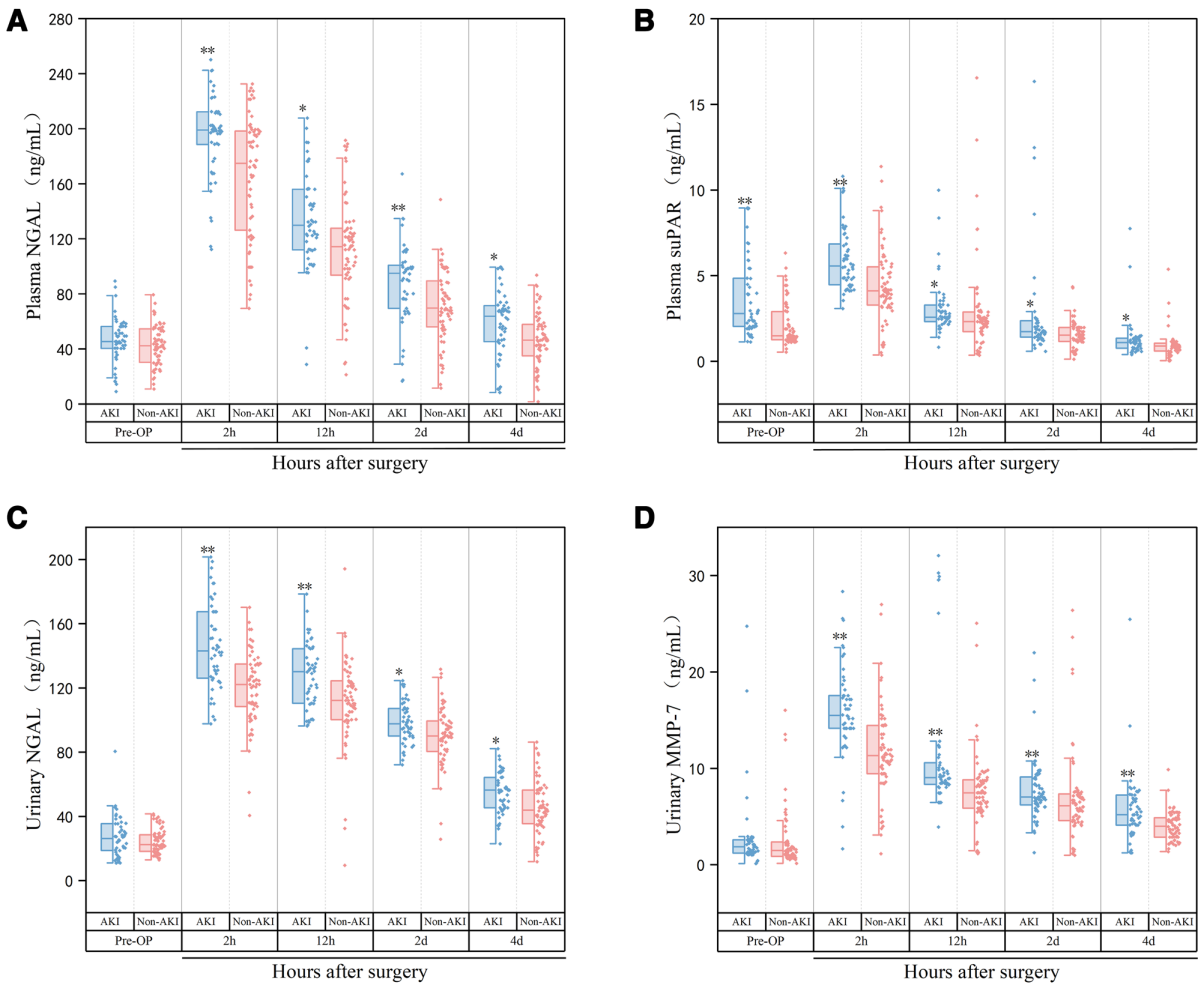
Dynamic changes of plasma and urinary biomarkers in patients who developed AKI. Serial measurements of (A) plasma NGAL, (B) plasma suPAR, (C) urinary NGAL, and (D) urinary MMP-7 levels in AKI and non-AKI patients at Pre-op, 2 hours, 12 hours, 2 days, and 4 days postoperatively. Data are presented as box (IQR) and scatter (individual values) plots, with the central line in the box indicating the median. Blue and red represent the AKI and non-AKI groups, respectively. Statistical comparisons were performed using the Mann–Whitney *U* test (*P < .05; **P < .01, AKI vs non-AKI at each time point). AKI = acute kidney injury, IQR = interquartile range, MMP-7 = matrix metalloproteinase-7, NGAL = neutrophil gelatinase-associated lipocalin, suPAR = soluble urokinase-type plasminogen activator receptor.

The perioperative dynamic changes of each biomarker in the 2 groups are shown in (Fig. 2) and (Table 2). Scr levels in the AKI group were significantly higher than those in the non-AKI group at 2 and 4 days postoperatively (*P* < .001). Notably, preceding the significant difference in Scr, plasma suPAR levels were already elevated in the AKI group compared to the non-AKI group as early as the preoperative period (*P* < .001), and this significant difference persisted at all postoperative time points (*P* < .05). At every measured postoperative time point, the levels of all biomarkers were significantly higher in the AKI group than in the non-AKI group (Fig. 2). The peak concentrations for all biomarkers in the AKI group occurred at 2 hours postoperatively. At this specific time point, levels in the AKI group were significantly higher than those in the non-AKI group *(P* < .001). Although the levels gradually declined thereafter, they remained statistically significantly higher in the AKI group throughout the entire observation period (all *P* < .05). As shown in (Fig. S1, Supplemental Digital Content 1), the predictive value of each biomarker for CSA-AKI was evaluated using receiver operating characteristic curve analysis. The results demonstrated that preoperative plasma suPAR exhibited good predictive ability (AUC = 0.727). At 2 hours postoperatively, plasma NGAL (AUC = 0.707), urinary NGAL (AUC = 0.807), and urinary MMP-7 (AUC = 0.805) all reached their optimal predictive performance.

**Table 2:** Serial biomarker levels in AKI versus non-AKI groups.

	AKI	Non-AKI	*Z*	*P*
Scr (μmol/L)				
Pre-op	69 (54, 91)	68 (59, 91)	0.256	.8
2 h	75 (67, 109)	83 (72, 99)	0.956	.34
12 h	95 (84, 144)	94 (78, 108)	−1.594	.111
2 d	167 (121, 288)	98 (69, 114)	−6.330	<.001
4 d	156 (111, 269)	83 (61, 112)	−6.152	<.001
NGAL (ng/mL)				
Preop	45.421 (40.421, 56.321)	41.741 (30.337, 54.625)	−1.394	.163
2 h	198.644 (188.535, 212.242)	173.370 (126.254, 198.321)	−3.808	<.001
12 h	129.236 (112.047, 156.000)	113.840 (93.688, 127.699)	−3.142	.002
2 d	94.505 (69.468, 100.780)	69.456 (55.976, 89.456)	−3.275	.001
4 d	62.765 (45.346, 71.455)	46.326 (35.000, 57.875)	−3.038	.002
suPAR (ng/mL)				
Pre-op	2.325 (1.907, 2.802)	1.448 (1.255, 2.022)	−4.181	<.001
2 h	5.518 (4.448, 6.749)	4.155 (3.213, 5.516)	−4.025	<.001
12 h	2.576 (2.321, 3.279)	2.321 (1.729, 2.879)	−2.751	.006
2 d	1.720 (1.412, 2.368)	1.521 (1.152, 1.978)	−2.592	.010
4 d	1.084 (0.757, 1.349)	0.881 (0.597, 1.057)	−3.250	.001
uNGAL (ng/mL)				
Pre-op	27.254 (18.740, 38.544)	19.433 (16.412, 27.155)	−2.715	.007
2 h	142.156 (124.979, 167.412)	115.432 (101.128, 129.824)	−5.642	<.001
12 h	129.880 (110.433, 144.643)	112.152 (100.233, 124.403)	−3.462	.001
2 d	97.038 (89.989, 107.140)	89.990 (80.416, 99.416)	−2.790	.005
4 d	56.321 (45.432, 64.308)	43.421 (35.432, 56.432)	−3.206	.001
uMMP-7 (ng/mL)				
Pre-op	1.837 (1.200, 2.578)	1.450 (0.859, 2.354)	−1.653	.098
2 h	16.546 (14.156, 21.156)	11.248 (9.456, 14.456)	−5.608	<.001
12 h	8.966 (8.330, 10.590)	7.456 (5.871, 8.825)	−4.443	<.001
2 d	7.562 (6.684, 9.425)	6.014 (4.574, 7.340)	−3.808	<.001
4 d	5.184 (4.116, 7.239)	3.957 (2.869, 4.873)	−3.635	<.001

Data are expressed as median (IQR). Group comparisons were conducted using the Mann–Whitney *U* test, with *Z* and *P* values indicating statistical significance.

AKI = acute kidney injury, suPAR = soluble urokinase-type plasminogen activator receptor, uMMP-7 = urinary matrix metalloproteinase-7, uNGAL = urinary neutrophil gelatinase-associated lipocalin.

Time points: 12 h = postoperative 12-hour, 2 d = postoperative 2-day, 2 h = postoperative 2-hour, 4 d = postoperative 4-day, Pre-op = preoperative.

### 3.3. Logistic regression analysis

Multivariable binary logistic regression analysis was performed, incorporating clinical risk factors and early biomarkers (suPAR, NGAL, and MMP-7). After adjusting for relevant covariates, the results demonstrated significant associations between the following variables and the incidence of CSA-AKI: CPB time (odds ratio [OR] = 1.02, 95% confidence interval [CI]: 1.01–1.03, *P* = .002), preoperative plasma suPAR (OR = 1.87, 95% CI: 1.07–3.29, *P* = .028), postoperative 2 hours urinary NGAL (OR = 1.06, 95% CI: 1.04–1.10, *P* < .001), and postoperative 2 hours urinary MMP-7 (OR = 1.26, 95% CI: 1.12–1.46, *P* < .001). These findings identify CPB time, preoperative plasma suPAR, postoperative 2 hours urinary NGAL, and postoperative 2 hours urinary MMP-7 as independent predictors of CSA-AKI. The complete results are summarized in (Table 3).

**Table 3:** Univariate and multivariate analysis of risk factors for CSA-AKI.

Variables	Univariate analysis		Multivariate analysis	
	*P* value	OR (95% CI)	*P* value	OR (95% CI)
Age	.571	0.99 (0.96–1.02)		
BMI	.484	0.97 (0.91–1.05)		
Emergency	.008	2.80 (1.31–5.99)	-	-
WBC	.007	1.13 (1.03–1.24)	-	-
Hb	.655	1.00 (0.98–1.02)		
ALB	.478	0.98 (0.91–1.05)		
Scr	.59	1.00 (0.99–1.01)		
eGFR	.865	1.00 (0.99–1.01)		
Blood loss	.057	1.00 (1.00–1.00)		
RBC Transfusion	.082	1.11 (0.99–1.25)		
Operation time	.002	1.01 (1.00–1.01)	-	-
CPB time	<.001	1.02 (1.01–1.03)	.002	1.02 (1.01–1.03)
suPAR Pre-op	.013	1.69 (1.12–2.57)	.028	1.87 (1.07–3.29)
pNGAL 2 h	<.001	1.02 (1.01–1.03)	-	-
uNGAL 2 h	<.001	1.06 (1.03–1.08)	<.001	1.06 (1.04–1.10)
uMMP-7 2 h	<.001	1.23 (1.12–1.36)	<.001	1.26 (1.12–1.46)

Variables with *P* < .05 in the univariate analysis were initially entered into the multivariable logistic regression. Dashes (-) represent variables that were eliminated during the stepwise selection process.

ALB = albumin, BMI = body mass index, CI = confidence interval, CPB = cardiopulmonary bypass, eGFR = estimated glomerular filtration rate, Hb = hemoglobin, OR = odds ratio, RBC = red blood cell, Scr = serum creatinine, suPAR = soluble urokinase-type plasminogen activator receptor, uMMP-7 = urinary matrix metalloproteinase-7, uNGAL = urinary neutrophil gelatinase-associated lipocalin, WBC = white blood cell.

### 3.4. Predictive value of combined detection for CSA-AKI

This study demonstrates the enhanced predictive capability of combining novel biomarkers with clinical risk factors through 3 progressively developed models. The strategic integration of these parameters significantly improves early identification of patients at high risk for CSA-AKI.

Model composition and development

- Model 1 (Mod1): Postoperative 2 hours uNGAL + Postoperative 2 hours uMMP-7
- Model 2 (Mod2): Preoperative suPAR + Postoperative 2 hours uNGAL + Postoperative 2 hours uMMP-7
- Model 3 (Mod3): Mod2 + CPB time

**Figure 3.**
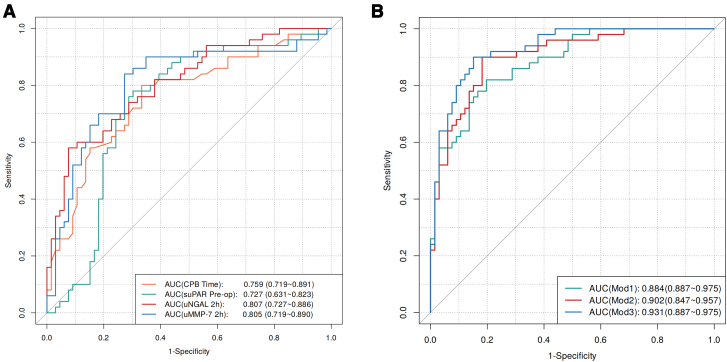
(A) Diagnostic power of the biomarkers for CSA-AKI. (B) ROC curves illustrate the predictive performance of different models for CSA-AKI. The models are defined as follows: Mod1: combination of postoperative 2 hours uNGAL and uMMP-7; Mod2: combination of preoperative plasma suPAR, postoperative 2 hours uNGAL, and uMMP-7; and Mod3: combination of model 2 variables and CPB time. AUC = area under the curve, CPB = cardiopulmonary bypass, CSA-AKI = cardiac surgery-associated acute kidney injury, Pre-op = preoperative, ROC = receiver operating characteristic, suPAR = soluble urokinase-type plasminogen activator receptor, uMMP-7 = urinary matrix metalloproteinase-7, uNGAL = urinary neutrophil gelatinase-associated lipocalin.

The combination of multiple biomarkers substantially improved predictive performance. Specifically, the combination of suPAR, uNGAL, and uMMP-7 (Mod2) achieved an AUC of 0.902, with a sensitivity of 90.0% and a specificity of 81.8%. Further incorporation of CPB time (Mod3) increased the AUC to 0.931, representing the highest diagnostic accuracy in this integrated biomarker-clinical factor analysis (Fig. 3). The optimal cutoff values, positive predictive value, and negative predictive value for each parameter and model are presented in (Table 4).

**Table 4:** Diagnostic value of various parameters and models for CSA-AKI.

Parameter/model	AUC	Sensitivity	Specificity	Youden index	Cutoff value	PPV	NPV
CPB time	0.759	0.800	0.667	0.467	168.000	0.645	0.815
suPAR Pre-op	0.727	0.780	0.697	0.477	1.897	0.661	0.807
uNGAL 2 h	0.807	0.580	0.924	0.504	140.188	0.853	0.744
uMMP-7 2 h	0.805	0.840	0.727	0.567	14.151	0.700	0.857
Mod1	0.884	0.820	0.803	0.623	0.397	0.759	0.855
Mod2	0.902	0.900	0.818	0.718	0.387	0.790	0.915
Mod3	0.931	0.900	0.848	0.748	0.342	0.818	0.918

Footnotes: Mod2 includes preoperative suPAR, postoperative 2 hours uNGAL, and postoperative 2 hours uMMP-7. Mod3 includes the predictors in Mod2 plus CPB time. For the definition of Mod1, refer to the Section 2.

AUC = area under the curve, CPB = cardiopulmonary bypass, MMP-7 = matrix metalloproteinase-7, NPV = negative predictive value (the optimal cutoff value was determined by maximizing Youden index), Postop = postoperative, PPV = positive predictive value, Pre-op = preoperative, suPAR = soluble urokinase-type plasminogen activator receptor, uNGAL = urinary neutrophil gelatinase-associated lipocalin.

**Figure 4.**
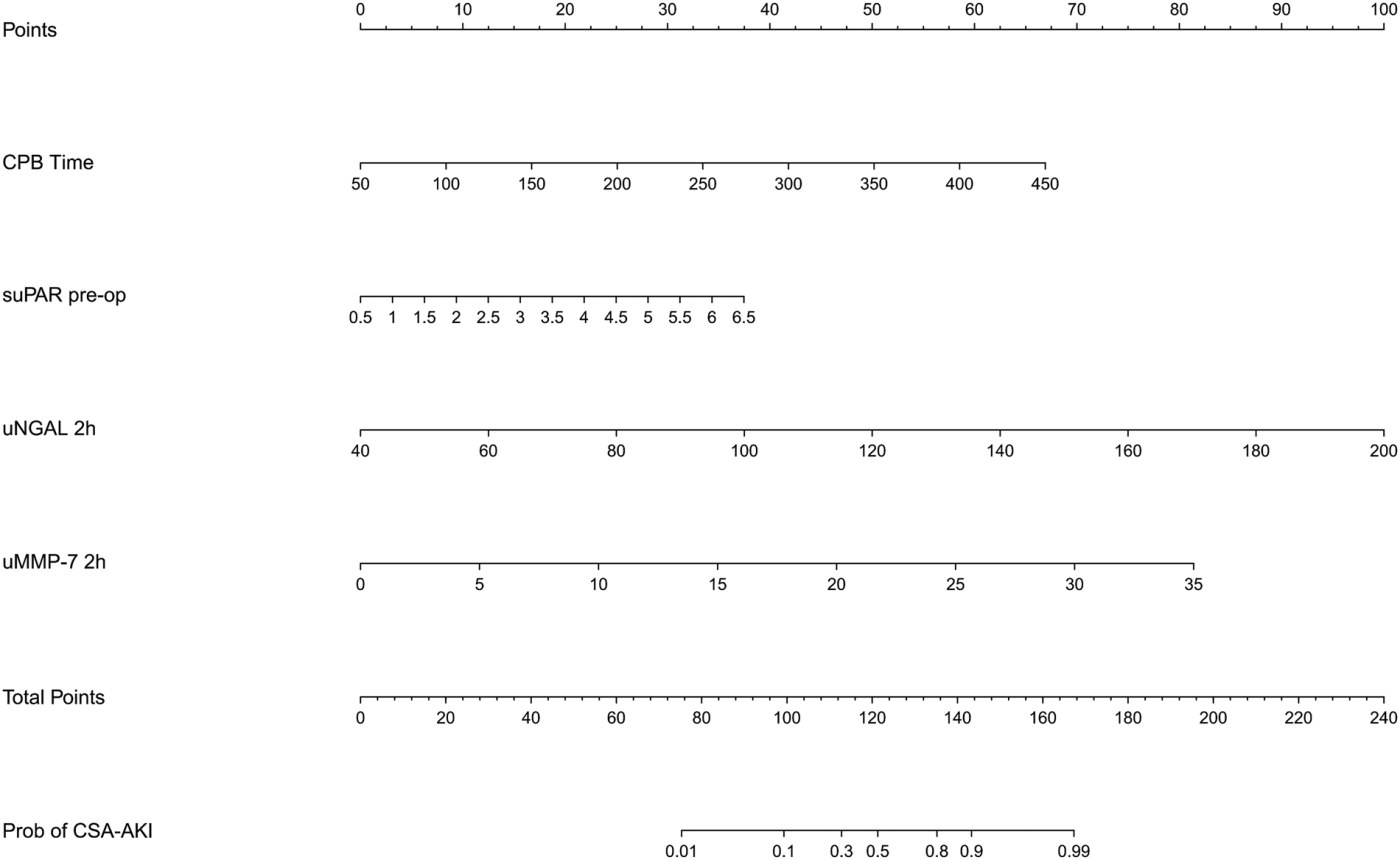
Nomogram for CSA-AKI. Scores assigned to different variables in the nomogram determine the risk of developing CSA-AKI. CPB = cardiopulmonary bypass, CSA-AKI = cardiac surgery-associated acute kidney injury, Pre-op = preoperative, suPAR = soluble urokinase-type plasminogen activator receptor, uMMP-7, urinary matrix metalloproteinase-7, uNGAL = urinary neutrophil gelatinase-associated lipocalin,.

Based on the results of the above multivariate analysis, the complete logistic regression equation for predicting the probability of CSA-AKI is as follows: Logit(*P*) = ln[*P*/(1 − *P*)] = −15.76 + (0.017 × CPB time) + (0.624 × preoperative plasma suPAR concentration) + (0.062 × postoperative 2 hours urinary NGAL concentration) + (0.232 × postoperative 2 hours urinary MMP-7 concentration). Where *P* represents the predicted probability of a patient developing CSA-AKI. The nomogram corresponding to this equation is shown in (Fig. 4).

### 3.5. Development of the predictive nomogram

Based on the results derived from multivariable logistic regression analysis, 4 key independent predictors were identified and incorporated into the model: CPB time, preoperative suPAR, postoperative 2 hours uNGAL, and postoperative 2 hours uMMP-7. These variables were programmed into R 4.2.3 using the rms package (regression modeling strategies) to construct a nomogram for predicting the risk of acute kidney injury following CPB cardiac surgery (Fig. 4).

### 3.6. Validation of the nomogram

**Figure 5.**
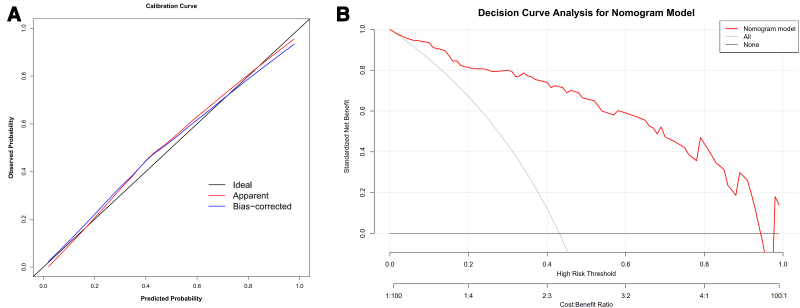
(A) Calibration curve of the nomogram in the nomogram prediction model. The calibration curve illustrates the agreement between predicted risks (*X*-axis) and actual outcomes (*Y*-axis) for the nomogram. (B) The DCA for the prediction model. The DCA demonstrates that the nomogram enhances net benefits and encompasses a wide range of threshold probabilities in predicting the occurrence of CSA-AKI. CSA-AKI = cardiac surgery-associated acute kidney injury, DCA = decision curve analysis.

The calibration curve for the nomogram demonstrated excellent agreement between predicted and observed probabilities of AKI (Fig. 5A). The bias-corrected curve closely followed the ideal reference line, indicating robust predictive accuracy. Decision curve analysis confirmed the clinical utility of the model across a wide range of threshold probabilities (Fig. 5B). The nomogram provided superior net benefit compared to default strategies, showing positive clinical value when intervention thresholds ranged from 10% to 80%. These results validate both the statistical accuracy and clinical applicability of the prediction model for risk stratification in cardiac surgery patients.

## 4. Discussion

In this observational cohort study, we evaluated the incidence of CSA-AKI according to KDIGO criteria and developed a predictive model by combining biomarkers with clinical risk factors. Among the 116 enrolled patients, 50 developed postoperative AKI, of whom 4% in the AKI group required RRT. According to relevant studies, the incidence of AKI in general cardiovascular surgery patients is approximately 30%.^[24,25]^ The higher incidence of AKI observed in this study may be attributed to the complexity of the included surgical types (such as combined CABG and valve replacement surgery, aortic dissection surgery, etc). Patients with AKI demonstrated significantly higher in-hospital mortality than non-AKI patients (*P* = .008). Multivariate analysis identified CPB time, preoperative plasma suPAR, postoperative 2 hours urinary NGAL, and postoperative 2 hours urinary MMP-7 as independent predictors of CSA-AKI. Prolonged CPB time significantly increased AKI risk, consistent with most literature reports,^[26,27]^ though controversies persist across studies, potentially due to population heterogeneity and insufficient adjustment for confounding factors.^[28,29]^ Furthermore, a statistically significant difference was observed in intraoperative RBC transfusion volume between the 2 patient groups (*P* = .028). Univariate analysis indicated that preoperative WBC count was associated with AKI occurrence^[30–34]^; however, it did not reach independent significance in multivariate analysis, potentially due to the relatively small sample size. Further validation with an expanded cohort is warranted. Just et al reported^[24]^ that emergency-admission patients had a higher incidence of AKI (*P* = .007), which aligns with our findings. We recommend optimizing surgical techniques and enhancing team proficiency to rationally control CPB time, thereby reducing the risk of AKI.

Postoperative 2-hour urinary NGAL levels were significantly elevated in the AKI group, achieving an AUC of 0.807, which outperformed contemporaneous plasma NGAL measurements and aligned with previous studies,^[35,36]^ suggesting its potential superiority for early prediction. However, controversies persist regarding NGAL’s performance in adult populations and the comparative value of urinary versus plasma NGAL. Some studies reported plasma NGAL correlated with AKI severity while urinary NGAL showed no significant difference,^[37]^ and GFR may influence plasma NGAL levels.^[38]^ Additionally, the peak timing of urinary NGAL in AKI patients remains undetermined, necessitating further large-scale multicenter validation.

This study investigated the predictive value of plasma suPAR for AKI following CPB cardiac surgery. The results demonstrated that plasma suPAR levels were significantly elevated in the AKI group compared to the non-AKI group at all time points, including preoperative and postoperative measurements, indicating a close association between suPAR and CPB-associated AKI. Preoperative plasma suPAR (AUC = 0.727, sensitivity 78%, and specificity 69.7%) exhibited superior predictive performance compared to measurements taken at any postoperative time point. These findings are consistent with previous studies supporting the correlation between preoperative suPAR levels and both the occurrence and severity of AKI.^[39]^Furthermore, suPAR showed a significant correlation with preoperative WBC count, suggesting its involvement in inflammatory mechanisms. Beyond its diagnostic value, suPAR may also predict renal recovery, as patients with lower levels demonstrated a higher likelihood of regained renal function.^[15]^ Targeting suPAR offers novel therapeutic perspectives for AKI prevention and treatment.^[16]^ However, the clinical application of suPAR remains limited, necessitating further validation through multicenter, large-sample studies to advance personalized risk assessment and preventive strategies.

The results of this study demonstrated that urinary MMP-7 levels were significantly elevated in the AKI group at all postoperative time points compared to the non-AKI group, peaking at 2 hours postsurgery (AUC = 0.805, sensitivity 84.0%, and specificity 72.7%). Its predictive performance was comparable to that of contemporaneous urinary NGAL (AUC = 0.807, sensitivity 58.0%, and specificity 92.4%), with higher sensitivity. Urinary MMP-7, a zinc-dependent endopeptidase, is primarily expressed in renal tubular epithelial cells. It is nearly undetectable in healthy renal tissue but significantly upregulated in AKI and CKD states.^[22,40]^ Its expression is regulated by the Wnt/β-catenin signaling pathway and may exert tubular protection and regeneration in early AKI through degradation of Fas ligand and activation of β-catenin, though it may also contribute to fibrotic processes in later stages.^[41]^ Multiple clinical studies support urinary MMP-7 as an early predictive biomarker for AKI following cardiac surgery. A multicenter study encompassing both pediatric and adult populations reported AUC values of 0.81 and 0.76 for predicting severe AKI, respectively, outperforming biomarkers such as NGAL, interleukin-18, and the combined biomarker tissue inhibitor of metalloproteinase-2 × insulin-like growth factor binding protein-7,^[42]^ highlighting its strong potential for clinical application.

This study evaluated the predictive value of combining multiple biomarkers with clinical risk factors for early detection of CSA-AKI. The integration of preoperative plasma suPAR, postoperative 2 hours urinary NGAL, and urinary MMP-7 achieved an AUC of 0.902, demonstrating excellent diagnostic capability. This multi-marker strategy encompasses the complete injury process, including systemic inflammation activation, tubular damage, and pathway dysregulation. It identifies high-risk patients 24 to 48 hours earlier than traditional creatinine-based diagnosis, providing a quantitative tool for KDIGO-based stratified management. Further incorporation of CPB time into the model enhanced predictive performance, elevating the AUC to 0.931.While most existing studies focus on conventional risk factor combinations,^[43]^ this work pioneers the synergistic use of biomarkers with intraoperative variables. CPB time exceeding 168 minutes serves as a critical early warning indicator for high-risk AKI following cardiac surgery. Prolonged surgical duration demonstrates a cumulative risk effect, with each additional 10 minutes of CPB time increasing AKI risk by approximately 22%. Despite the superior performance of this multidimensional approach, its clinical applicability requires further validation through large-scale studies to clarify its utility in multi-timepoint and multidimensional assessment systems.

Regarding the clinical application of the predictive model, it is essential to clarify that our study utilizes different thresholds derived for different purposes. As reported in Table 4, the cutoff value for Model 3 is 0.342, which was derived by maximizing the Youden index. This threshold is intended for maximizing diagnostic classification by balancing sensitivity and specificity. In contrast, practical clinical decision-making is based on optimizing net benefit rather than mathematical diagnostic accuracy. Therefore, based on the results of the decision curve analysis, we recommend setting a predicted probability ≥45% as the critical threshold for initiating renal protective strategies. When the model classifies a patient as high-risk, a standardized intervention protocol should be initiated immediately, specifically including: optimizing hemodynamic management to maintain a mean arterial pressure (MAP) ≥65 mm Hg; strictly avoiding nephrotoxic drugs; implementing goal-directed fluid resuscitation to ensure renal perfusion; and closely monitoring renal function and hourly urine output. This model may support early risk stratification and help identify high-risk patients from the intensive care population. However, because this is a single-center observational study with only internal validation, the nomogram is not ready for immediate clinical deployment. Further external validation in multicenter cohorts is required before this early warning pathway can be routinely implemented to guide renal protective measures.

This study has several limitations that should be acknowledged. First, as a single-center observational study with a relatively small sample size, only internal validation was performed without external validation. Second, subgroup analyses for moderate-to-severe acute kidney injury and long-term patient outcome follow-up were not conducted. Third, urinary biomarker concentrations were not normalized to urinary creatinine, and some perioperative confounding factors, including fluid management, hemodynamic fluctuations, and exposure to nephrotoxic medications, were not incorporated into the predictive model, which may limit the comprehensiveness of the findings. In addition, patients who died or experienced cardiac arrest within 48 hours postoperatively were excluded, which may introduce a degree of survivorship bias; consequently, the true incidence of CSA-AKI might be slightly higher than the observed rate of 43.1%. Nevertheless, only 3 patients were excluded due to early postoperative death, suggesting that the impact of such bias is minimal and acceptable for an exploratory single-center study. Despite the above limitations, the combined model incorporating multiple biomarkers and clinical variables demonstrated excellent early predictive performance for CSA-AKI. Future multicenter, large-scale studies are warranted to externally validate these findings and further refine risk stratification and early renal-protective intervention strategies.

## 5. Conclusion

The combined detection of preoperative plasma suPAR, postoperative 2 hours urinary NGAL, and urinary MMP-7 significantly enhances the diagnostic efficacy for CSA-AKI. Further integration of CPB time into a nomogram-based predictive model provides more accurate early warning capabilities, facilitating timely initiation of renal protective measures. However, external validation in multicenter cohorts is required before this early warning pathway can be routinely implemented in clinical practice.

## Acknowledgments

We wish to express our sincere appreciation to the investigators at our clinical site and to the patients who participated in this trial for their essential contributions.

## Author contributions

**Formal analysis:** Yuhao Liang.

**Funding acquisition:** Jie Hu.

**Investigation:** Xuanxuan Xu.

**Resources:** Huiling Si, Axue Cheng, Junlong Wang, Tao Liu, Hui Wang, Jie Hu.

**Software:** Shuaibo Shi.

**Supervision:** Tao Liu, Jie Hu.

**Visualization:** Shuaibo Shi, Huiling Si.

**Writing – original draft:** Xuanxuan Xu, Yuhao Liang.

**Writing – review & editing:** Xuanxuan Xu, Yuhao Liang, Shuaibo Shi, Huiling Si, Axue Cheng, Junlong Wang, Tao Liu, Hui Wang, Jie Hu.



## References

[1] Rosner MH Okusa MD. Acute kidney injury associated with cardiac surgery. Clin J Am Soc Nephrol. 2006;1:19–32. 17699187 10.2215/CJN.00240605

[2] Hoste EAJ Bagshaw SM Bellomo R. Epidemiology of acute kidney injury in critically ill patients: the multinational AKI-EPI study. Intensive Care Med. 2015;41:1411–23. 26162677 10.1007/s00134-015-3934-7

[3] Mishra PK Luckraz H Nandi J. Long-term quality of life postacute kidney injury in cardiac surgery patients. Ann Card Anaesth. 2018;21:41–5. 29336390 10.4103/aca.ACA_104_17 PMC5791486

[4] Bellomo R Auriemma S Fabbri A. The pathophysiology of cardiac surgery-associated acute kidney injury (CSA-AKI). Int J Artif Organs. 2008;31:166–78. 18311733 10.1177/039139880803100210

[5] Khwaja A. KDIGO clinical practice guidelines for acute kidney injury. Nephron Clin Pract. 2012;120:c179–184. 22890468 10.1159/000339789

[6] Lin J Fernandez H Shashaty MG. False-positive rate of AKI using consensus creatinine-based criteria. Clin J Am Soc Nephrol. 2015;10:1723–31. 26336912 10.2215/CJN.02430315 PMC4594067

[7] Flo TH Smith KD Sato S. Lipocalin 2 mediates an innate immune response to bacterial infection by sequestrating iron. Nature. 2004;432:917–21. 15531878 10.1038/nature03104

[8] Marakala V. Neutrophil gelatinase-associated lipocalin (NGAL) in kidney injury – a systematic review. Clin Chim Acta. 2022;536:135–41. 36150522 10.1016/j.cca.2022.08.029

[9] Uzawa A Kojima Y Ozawa Y. Serum level of soluble urokinase plasminogen activator receptor (suPAR) as a disease severity marker of myasthenia gravis: a pilot study. Clin Exp Immunol. 2020;202:321–4. 32706905 10.1111/cei.13499 PMC7670129

[10] Rasmussen LJH Petersen JEV Eugen-Olsen J. Soluble urokinase plasminogen activator receptor (suPAR) as a biomarker of systemic chronic inflammation. Front Immunol. 2021;12:780641. 34925360 10.3389/fimmu.2021.780641 PMC8674945

[11] Şirinoğlu M Soysal A Karaaslan A. The diagnostic value of soluble urokinase plasminogen activator receptor (suPAR) compared to C-reactive protein (CRP) and procalcitonin (PCT) in children with systemic inflammatory response syndrome (SIRS). J Infect Chemother. 2017;23:17–22. 27771157 10.1016/j.jiac.2016.08.015

[12] Su L Zhang J Peng Z. The role of kidney injury biomarkers in COVID-19. Ren Fail. 2022;44:1281–9. 10.1080/0886022X.2022.2107544 PMC9359166 35930243

[13] Huang Y Huang S Zhuo X Lin M. Predictive value of suPAR in AKI: a systematic review and meta-analysis. Clin Exp Nephrol. 2023;27:1–11. 36469196 10.1007/s10157-022-02300-2 PMC9734903

[14] Hall A Crichton S Varrier M Bear DE Ostermann M. suPAR as a marker of infection in acute kidney injury – a prospective observational study. BMC Nephrol. 2018;19:191. 30071826 10.1186/s12882-018-0990-6 PMC6090935

[15] Mossanen J Pracht J Jansen T. Elevated soluble urokinase plasminogen activator receptor and proenkephalin serum levels predict the development of acute kidney injury after cardiac surgery. Int J Mol Sci . 2017;18:1662. 28758975 10.3390/ijms18081662 PMC5578052

[16] Hayek SS Leaf DE Samman Tahhan A. Soluble urokinase receptor and acute kidney injury. N Engl J Med. 2020;382:416–26. 31995687 10.1056/NEJMoa1911481 PMC7065830

[17] Jankowski L Pruc M Gasecka A. A comprehensive review and meta-analysis of suPAR as a predictor of acute kidney injury. Ann Agric Environ Med. 2023;30:364–8. 37387388 10.26444/aaem/167464

[18] Gungor AB Pennathur P Guran HS Chu W Roshanov PS. Soluble urokinase plasminogen activator receptor and perioperative complications: a systematic review. BMC Anesthesiol. 2025;25:558. 41233776 10.1186/s12871-025-03350-1 PMC12613401

[19] Liu Z Tan RJ Liu Y. The many faces of matrix metalloproteinase-7 in kidney diseases. Biomolecules. 2020;10:960. 32630493 10.3390/biom10060960 PMC7356035

[20] Gaide Chevronnay HP Selvais C Emonard H Galant C Marbaix E Henriet P. Regulation of matrix metalloproteinases activity studied in human endometrium as a paradigm of cyclic tissue breakdown and regeneration. Biochim Biophys Acta. 2012;1824:146–56. 21982799 10.1016/j.bbapap.2011.09.003

[21] He W Tan RJ Li Y. Matrix metalloproteinase-7 as a surrogate marker predicts renal wnt/β-catenin activity in CKD. J Am Soc Nephrol. 2012;23:294–304. 22095947 10.1681/ASN.2011050490 PMC3269179

[22] Xiao L Zhou D Tan RJ. Sustained activation of wnt/β-catenin signaling drives AKI to CKD progression. J Am Soc Nephrol. 2016;27:1727–40. 26453613 10.1681/ASN.2015040449 PMC4884114

[23] Wozniak J Floege J Ostendorf T Ludwig A. Key metalloproteinase-mediated pathways in the kidney. Nat Rev Nephrol. 2021;17:513–27. 33879883 10.1038/s41581-021-00415-5

[24] Just IA Alborzi F Godde M. Cardiac surgery-related acute kidney injury risk factors, clinical course, management suggestions. J Cardiothorac Vasc Anesth. 2022;36:444–51. 34130896 10.1053/j.jvca.2021.05.012

[25] Czerny M Grabenwöger M Berger T. EACTS/STS guidelines for diagnosing and treating acute and chronic syndromes of the aortic organ. Ann Thorac Surg. 2024;118:5–115. 38416090 10.1016/j.athoracsur.2024.01.021

[26] Xu SJ Liu J Li L. Cardiopulmonary bypass time is an independent risk factor for acute kidney injury in emergent thoracic aortic surgery: a retrospective cohort study. J Cardiothorac Surg. 2019;14:90. 31064409 10.1186/s13019-019-0907-x PMC6505293

[27] Zhang K Shang J Chen Y Huo Y Li B Hu Z. The prognosis and risk factors for acute kidney injury in high-risk patients after surgery for type a aortic dissection in the ICU. J Thorac Dis. 2021;13:4427–37. 34422369 10.21037/jtd-21-823 PMC8339792

[28] Kowalik MM Lango R Klajbor K. Incidence- and mortality-related risk factors of acute kidney injury requiring hemofiltration treatment in patients undergoing cardiac surgery: a single-center 6-year experience. J Cardiothorac Vasc Anesth. 2011;25:619–24. 21354827 10.1053/j.jvca.2010.12.011

[29] Kim WH Lee SM Choi JW. Simplified clinical risk score to predict acute kidney injury after aortic surgery. J Cardiothorac Vasc Anesth. 2013;27:1158–66. 24050856 10.1053/j.jvca.2013.04.007

[30] Dimopoulos S Zagkotsis G Kinti C. Incidence and peri-operative risk factors for development of acute kidney injury in patients after cardiac surgery: a prospective observational study. World J Clin Cases. 2023;11:3791–801. 37383133 10.12998/wjcc.v11.i16.3791 PMC10294155

[31] Ryugo M Monta O Saitou S. Risk analysis of acute kidney injury after cardiac surgery and protective effect by less invasive surgery. Kyobu Geka. 2020;73:895–900. 33130709

[32] Husain-Syed F Quattrone MG Ferrari F. Clinical and operative determinants of acute kidney injury after cardiac surgery. Cardiorenal Med. 2020;10:340–52. 32599584 10.1159/000507777

[33] Khan UA Coca SG Hong K. Blood transfusions are associated with urinary biomarkers of kidney injury in cardiac surgery. J Thorac Cardiovasc Surg. 2014;148:726–32. 24820190 10.1016/j.jtcvs.2013.09.080 PMC4104243

[34] Oprea AD Del Rio JM Cooter M. Pre- and postoperative anemia, acute kidney injury, and mortality after coronary artery bypass grafting surgery: a retrospective observational study. Can J Anaesth. 2018;65:46–59. 29098634 10.1007/s12630-017-0991-0

[35] Mishra J Dent C Tarabishi R. Neutrophil gelatinase-associated lipocalin (NGAL) as a biomarker for acute renal injury after cardiac surgery. Lancet. 2005;365:1231–8. 15811456 10.1016/S0140-6736(05)74811-X

[36] Bennett M Dent CL Ma Q. Urine NGAL predicts severity of acute kidney injury after cardiac surgery: a prospective study. Clin J Am Soc Nephrol. 2008;3:665–73. 18337554 10.2215/CJN.04010907 PMC2386703

[37] Larstorp ACK Salvador CL Svensvik BA Klingenberg O Distante S. Neutrophil gelatinase-associated lipocalin (NGAL) and cystatin C are early biomarkers of acute kidney injury associated with cardiac surgery. Scand J Clin Lab Invest. 2022;82:410–8. 36036280 10.1080/00365513.2022.2114105

[38] Salvador CL Tøndel C Rowe AD. Renal function influences diagnostic markers in serum and urine: a study of guanidinoacetate, creatine, human epididymis protein 4, and neutrophil gelatinase–associated lipocalin in children. J Appl Lab Med. 2017;2:297–308. 33636843 10.1373/jalm.2016.022145

[39] Rasmussen SR Nielsen RV Møgelvang R Ostrowski SR Ravn HB. Prognostic value of suPAR and hsCRP on acute kidney injury after cardiac surgery. BMC Nephrol. 2021;22:120. 33827466 10.1186/s12882-021-02322-0 PMC8025450

[40] Zhou D Li Y Lin L Zhou L Igarashi P Liu Y. Tubule-specific ablation of endogenous β-catenin aggravates acute kidney injury in mice. Kidney Int. 2012;82:537–47. 22622501 10.1038/ki.2012.173 PMC3425732

[41] Fu H Zhou D Zhu H. Matrix metalloproteinase-7 protects against acute kidney injury by priming renal tubules for survival and regeneration. Kidney Int. 2019;95:1167–80. 30878215 10.1016/j.kint.2018.11.043 PMC6478554

[42] Yang X Chen C Teng S. Urinary matrix metalloproteinase-7 predicts severe AKI and poor outcomes after cardiac surgery. J Am Soc Nephrol. 2017;28:3373–82. 28698269 10.1681/ASN.2017020142 PMC5661292

[43] Jorge-Monjas P Bustamante-Munguira J Lorenzo M. Predicting cardiac surgery-associated acute kidney injury: the CRATE score. J Crit Care. 2016;31:130–8. 26700607 10.1016/j.jcrc.2015.11.004

