# Supplementary material for: Combining biomarkers with clinical risk factors to predict acute kidney injury after cardiopulmonary bypass surgery: An observational cohort study
Source: Medicine (Baltimore). 2026 Jul 10;105(28):e49765. doi: 10.1097/MD.0000000000049765 (PMC13363029; doi:10.1097/MD.0000000000049765)
Supplement: Supplementary file 1 [file medi-105-e49765-s001.docx]

*Supplementary Materials*

Fig 1S. ROC curves for predicting CSA-AKI based on perioperative biomarker levels. ROC curves demonstrate the discriminatory power of (A) plasma NGAL, (B) plasma suPAR, (C) urinary NGAL, and (D) urinary MMP-7 measured at Pre-op, Post-op 2h, Post-op 12h, Post-op 2d, and Post-op 4d after cardiac surgery. AUC values with 95% CI are indicated for each time point. The dashed black line represents the reference line of no discrimination (AUC = 0.5). Time points: Pre-op, preoperative; Post-op 2h, postoperative 2-hour; Post-op 12h, postoperative 12-hour; Post-op 2d, postoperative 2-day; Post-op 4d, postoperative 4-day; CI, confidence interval.
